# Association of cardiovascular health score trajectory and risk of subsequent cardiovascular disease in non-diabetic population: a cohort study

**DOI:** 10.1186/s12889-023-15569-z

**Published:** 2023-06-01

**Authors:** Hui Zhou, Xiong Ding, Shouling Wu, Jin Yan, Jianyun Cao

**Affiliations:** 1 Xiangya School of Nursing, Central South University, Changsha, Hunan China; 2 School of Public Health, Wuhan University, Wuhan, Hubei China; 3 Department of Cardiology, Kailuan General Hospital, Tangshan, Hebei China; 4 Nursing Department, The Third Xiangya Hospital, Central South University, Changsha, Hunan China; 5 Department of Reproductive Medicine, Xiangya Hospital, Central South University, Changsha, Hunan China

**Keywords:** Health behavior, Risk factors, Cardiovascular disease, Non-diabetic population, Longitudinal study

## Abstract

**Background:**

Diabetes is an important risk factor for cardiovascular disease (CVD), but in the non-diabetic population, high glucose values within the normal range are also positively associated with CVD risk. There is a lack of concern for people without diabetes and evidence is lacking regarding the association between changes in cardiovascular health score (CVHS) and CVD risk in the non-diabetic population.

**Methods:**

The current study included 37,970 non-diabetic participants free of CVD events in or before 2010 from the Kailuan Study and calculated CVHS according to the overall status of 7 cardiovascular health metrics between the 2006 and 2010 waves. Latent mixture models were used to explore the subgroups with different development trends included in the context of the Kailuan non-diabetic population and to identify the trajectory of each subgroup. The outcomes of the current study were CVD events, including myocardial infarction and stroke. CVHS trajectory was developed to predict subsequent CVD risk from 2010 to 2020. The Cox proportional hazard model was established to calculate the hazard ratios (HRs) and 95% confidence intervals (CIs) of CVD across different trajectory patterns.

**Results:**

Five distinct CVHS trajectory patterns were identified, including low-stable pattern (n = 2835), moderate-increasing pattern (n = 3492), moderate-decreasing pattern (n = 7526), high-stable I pattern (n = 17,135), and high-stable II pattern (n = 6982). Compared with the low-stable pattern, participants with the high-stable II pattern had a lower subsequent risk of CVD (HR = 0.22, 95%CI = 0.18–0.28); In stratification analysis, the lower risk for CVD was observed in females (HR = 0.10, 95%CI = 0.05–0.23, *P* for interaction < 0.05) and those aged < 60 years (HR = 0.16, 95%CI = 0.11 to 0.22, *P* for interaction < 0.05).

**Conclusions:**

CVHS trajectory patterns were associated with an altered CVD risk in the non-diabetic population. When stratified by age and sex, the association was stronger in young adults and females.

**Supplementary Information:**

The online version contains supplementary material available at 10.1186/s12889-023-15569-z.

## Background

Cardiovascular disease (CVD) is a leading cause of death and disability worldwide [1]. Although the long-time effort of health promotion organizations to improve healthy lifestyles has slowed down the development of the disease, leading to declines in crude and age-standardized mortality rates for CVD, the current prevalence of CVD in China plateaus or even increases [2].

Several guidelines have been published to highlight the importance of health behaviors and health factors for reducing CVD risk [3–5]. A randomized clinical trial also demonstrated that multi-domain lifestyle intervention, including changes in diet, increased physical activity, and management of metabolic risk factors, could reduce the risk of stroke and coronary heart disease [6]. In 2010, the American Heart Association introduced the construct of cardiovascular health, characterized by 4 health behaviors (smoking, body mass index, physical activity, and diet) and 3 health factors (blood cholesterol, blood pressure, and blood glucose) [7]. 7 metrics were each assigned 2 points for ideal, 1 for intermediate, and 0 for poor. The points were summed to yield a total cardiovascular health score ranging from 0 to 14 [8]. Emerging evidence focused on the association between CVD risk and cardiovascular health score (CVHS). In a Finnish study, higher adherence to the CVHS is associated with lower carotid lumen diameter and higher carotid distensibility [9]. The Framingham Offspring Study also showed that a better CVHS in midlife has salutary cardiometabolic benefits and may be associated with lower mortality later in life [10]. In addition, as the CVHS could change over time, how these changes were related to CVD was also explored (i.e., trajectory) [11, 12].

However, the association between change in CVHS and CVD risk is largely unknown among the non-diabetic population. To our knowledge, individuals with and without diabetes are at different risks for CVD outcomes that patients with diabetes had a 2- to 4-fold greater CVD risk compared to non-diabetic patients [13]. The hyperglycemia caused by diabetes may further increase other risk factors for CVD, such as elevated blood pressure, dyslipidemia, insulin resistance, and inflammatory response, and accelerate the atherosclerosis process [14]. The exclusion of the diabetic population may help to reduce the interference. While in the non-diabetic population, high glucose values within the normal range were also positively associated with the CVD risk [15, 16]. A better understanding of the risk factor burden that participants bear before diabetes diagnosis can enable us to identify those at greatest risk for the ultimate development of CVD [17]. Therefore, we analyzed data from 37,970 non-diabetic participants from the Kailuan Study to describe CVHS trajectory over four years and to investigate the association between CVHS trajectory and subsequent CVD risk.

## Methods

The data that support the findings of this study are available from the corresponding author upon reasonable request.

### Study population

The Kailuan Study is a population-based cohort study of employees and retirees recruited from the Kailuan Group, which is a large integrated group based on coal mines in Tangshan [18, 19]. In the Kailuan study, over 80% of the participants were men, and few female employees were underground coal miners or workers, which resulted in an imbalance between males and females. The present study was a retrospective analysis embedded in the Kailuan Study, comprising a standardized evaluation of cardiovascular risk factors in baseline and two subsequent follow-up waves. Briefly, starting from the 2006 wave, 101,510 participants were enrolled and underwent detailed questionnaire assessments, clinical examinations, and laboratory tests biennially, and they were followed annually to update their status on the clinical events (e.g., CVD). The CVHS trajectory was identified according to the change in CVHS between the 2006 wave and 2010 wave in non-diabetic participants with all seven cardiovascular health metrics at all time points to predict the CVD risk from 2010 to 2020. Of 49,013 participants without diabetes, we excluded 7919 participants missing the data of cardiovascular health metrics in any one of the follow up during the change assessment. Those participants who had known physician-diagnosed CVD events in or before 2010 (3124 participants) were also excluded. Finally, we explored the association between CVHS trajectory with subsequent CVD risk among the remaining 37,970 non-diabetic participants (Supplemental Figure S1). The characteristics of the participants included and excluded were shown in Supplemental Table S1. Additionally, we defined those who did not attend subsequent biennial health check-ups, and those who were not followed up until the outcome event occurred as the lost follow-up population, the loss up rate was 3.2% and the characteristics of them were displayed in Supplemental Table S2.

### Assessment of the CVHS and data collection

The individual cardiovascular health metrics are each assigned 2 points for ideal, 1 for intermediate, and 0 for poor according to the definitions promulgated by the American Heart Association [7] and modified feasibly for harmonization within the Kailuan Study [11, 12, 20]. Tobacco smoking was defined as self-reported never, past smoker, and current smoker. Body mass index was defined as < 24, 24–28, and ≥28 kg/m^2^ for the Chinese population [21], calculated as the weight in kilograms divided by height in meters squared. Physical activity was assessed according to no physical activity, 1–2 times per week and ≥ 20 min per session, and ≥ 3 times per week and ≥ 20 min per session. Considering the lack of healthy diet data in the Kailuan Study from 2006 to 2010 and the intense association between salt intake and the risk of CVD in the Chinese population, we applied salt intake as a surrogate measure and facilitated the quantification of salt intake in the questionnaire by asking participants to rate their habitual daily salt intake as low (< 6 g/d), moderate (6 to 10 g/d), or high (> 10 g/d), as described previously [11, 18]. Selection of total cholesterol < 200 mg/dL, blood pressure < 120/80 mmHg, and fasting blood glucose < 100 mg/dL as the optimal level, total cholesterol > 240 mg/dL or treated total cholesterol > 200 mg/dL, blood pressure > 140/90 mmHg, and fasting blood glucose > 126 mg/dL as the poor level, which is consistent with the definition of the American Heart Association [7]. The CVHS was on a 1–14 points scale in the current study with non-diabetic populations.

Information on demographic characteristics (e.g., age, sex, and education background), income level, smoking status, drinking status, physical activity, and salt intake were collected using a standardized questionnaire. Physical examinations (e.g., weight, height, blood pressure, and heart rate) were conducted by trained field workers. Laboratory tests, including total cholesterol, fasting blood glucose, high-sensitivity C-reactive protein, and serum creatinine, were assessed by an auto-analyzer (Hitachi 747; Hitachi) at the central laboratory of Kailuan General Hospital.

### Ascertainment of incident CVD events

The primary outcome was the first occurrence of a CVD event, comprised of myocardial infarction and stroke. Participants were monitored continuously from 2006 until the date of incident CVD diagnosis, death, or December 31, 2020. The CVD database was linked to the Municipal Social Insurance Institution and the Hospital Discharge Register, which covered all of the Kailuan Study participants, and was updated annually. Besides the history of CVD collected via a standard questionnaire, the International Classification of Diseases, Tenth Revision, codes were used to identify CVD cases (I21 for myocardial infarction; I60, I61, and I63 for stroke) [22], and an independent panel of physicians, blinded to the study design, reviewed the medical records for all suspected CVD events. Incident myocardial infarction was diagnosed according to the World Health Organization’s Multinational Monitoring of Trends and Determinants in Cardiovascular Disease criteria based on clinical symptoms and dynamic changes in cardiac enzymes and/or biomarker concentrations and electrocardiogram results [23, 24]. Stroke was diagnosed according to the World Health Organization criteria, based on signs, symptoms, neuroimages (from computed tomographic or magnetic resonance imaging), and other diagnostic reports [25, 26], as detailed previously. Mortality information was linked to the municipal death registries and checked annually against local residential records, with active confirmation of survival through subdistrict offices.

### Statistical analysis

The CVHS in 2006, 2008, and 2010 waves among non-diabetic participants were computed, and the trajectory patterns were identified by latent mixture modeling through the PROC TRAJ procedure [27, 28]. The best model fit with five patterns was assessed using the Bayesian information criterion, with the number of participants in each trajectory satisfied (> 5% of the overall population). Subject to the assumptions of proportional hazards being met, Cox proportional hazards regression was used to calculate hazard ratios (HRs) and 95% confidence intervals (CIs) of CVD risk along the CVHS trajectory from 2010 to 2020. Age and sex were adjusted in model 1; education background (primary, middle or high school, or college or above), income level (≥800 Chinese Yuan per month or < 800 Chinese Yuan per month) were further adjusted in model 2 on the basis of model 1; while model 3 included model 2 and drinking status (no, or yes), serum high-sensitivity C-reactive protein, estimated glomerular filtration rate (log-transformed), and heart rate for further adjustment. The incidence rates were calculated as the total number of events divided by the total person-years of follow-up and presented as events per 1000 person-years. We adopted 60 years as the cutoff for age according to the World Health Organization, and conducted stratification analysis by age (< 60 years, or ≥60 years) and sex (male, or female) to explore whether the potential association between the CVHS trajectory and CVD could be affected.

Several sensitivity analyses were also conducted. We adjusted CVHS in the 2006 wave and CVHS in the 2010 wave based on model 3, respectively, to further understand whether the association was independent of baseline cardiovascular health status. We conducted a sensitivity analysis by adjusting the antihypertensive medication and lipide-lowering medication due to their importance for the dynamics of the disease. We conducted a sensitivity analysis to explore the association between CVHS trajectory and CVD risk by excluding losing participants to check for the potential bias of losing to follow-up assessment. Analysis was also conducted to reduce the possibility of reverse causality by excluding incident CVD events onset during the first two years of follow-up. All statistical tests were 2-sided, and *P* < 0.05 was considered significant. Statistical analyses were performed using SAS, version 9.4 (SAS Institute Inc).

## Results

**Fig. 1:**
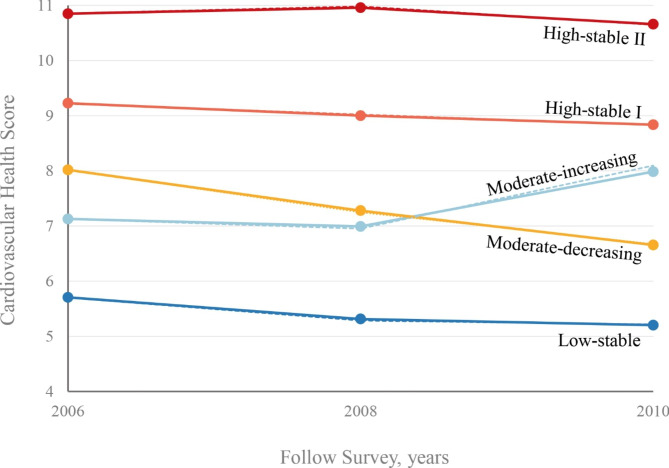
Five trajectory patterns of cardiovascular health score from 2006 to 2010

Five distinct trajectory patterns were identified among 37,970 non-diabetic participants based on the CVHS changes from 2006 to 2010 (Fig. 1): 7.5% (n = 2835) of participants who consistently had low-stable CVHS, 9.2% (n = 3492) of participants who started with moderate CVHS and experienced an increase, 19.8% (n = 7526) of participants who started with moderate CVHS and experienced a decrease, 45.1% (n = 17,135) of participants who had high-stable CVHS, and 18.4% (n = 6982) of participants who started with a relatively higher-stable CVHS (Table 1).

**Table 1:** Basic characteristics of participants according to the trajectory of CVHS from 2006 to 2010

	Low-stable	Moderate-increasing	Moderate-decreasing	High-stable I	High-stable II	*P* value
No. of participants	2835	3492	7526	17,135	6982	
Age, mean (SD), y	50.1±9.8	52.4±10.7	51.9±11.1	52.8±12.1	48.7±12.5	< 0.001
Men	2757(97.3)	3264(93.5)	6723(89.3)	13,186(77.0)	3038(43.5)	< 0.001
Heart rate, mean (SD), beats/min	75.9±10.5	73.9±10.4	74.0±10.1	72.6±9.9	71.2±9.1	< 0.001
Hs-CRP, median (IQR), mg/L	1.5(0.7–3.2)	1.2(0.5–2.8)	1.2(0.6–2.7)	1.0(0.4–2.2)	0.8(0.4–1.5)	< 0.001
eGFR,median(IQR),mL/min/1.73 m^2^	98.9(84.9-107.7)	87.3(72.5-102.4)	95.2(80.0-105.4)	90.0(74.2-103.2)	95.2(78.4-107.8)	< 0.001
CVHS in 2006, mean (SD), points	5.5±1.2	6.5±1.1	8.1±1.2	9.2±1.2	11.0±1.0	< 0.001
CVHS in 2010, mean (SD), points	5.1±1.3	8.4±1.0	6.3±1.0	8.8±1.2	10.8±1.1	< 0.001
Education background						< 0.001
Primary	218(7.7)	217(6.2)	577(7.67)	1067(6.23)	275(3.9)	
Middle/high school	2367(83.5)	3039(87.0)	6206(82.5)	14,314(83.5)	5157(73.9)	
College or above	250(8.8)	236(6.8)	743(9.9)	1754(10.2)	1550(22.2)	
Drinking status						< 0.001
No	981(34.6)	2166(62.0)	3510(46.6)	11,792(68.8)	5911(84.7)	
Yes	1854(65.4)	1326(38.0)	4016(53.4)	5343(31.2)	1071(15.3)	
Income level						< 0.001
< 800, Chinese yuan/mo	1371(48.4)	2053(58.8)	3388(45.0)	8469(49.4)	3334(47.8)	
≥ 800, Chinese yuan/mo	1464(51.6)	1439(41.2)	4138(55.0)	8666(50.6)	3648(52.3)	
Antihypertensive medications	1127(39.75)	903(25.9)	1976(26.3)	2468(14.4)	270(3.9)	< 0.001
Lipid-lowering medications	83(2.9)	59(1.7)	134(1.8)	149(0.9)	34(0.5)	< 0.001

Abbreviations: CVHS, cardiovascular health score; eGFR, estimated glomerular filtration rate; Hs-CRP, hypersensitive c-reactive protein

Data is n (%) or mean±SD or median (*P*_25_, *P*_75_), The Pearson chi-square test and ANOVA were used to compare differences between categorical variables and continuous variables between groups, respectively

During the follow-up period from 2010 to 2020, 1740 incident CVD events (of which 1421 were strokes, 339 were myocardial infarctions, and 20 were concurrent stroke and myocardial infarctions) were documented. The CVHS trajectory was significantly and inversely associated with CVD risk. The high-stable II pattern had the lowest risk of developing CVD events (Table 2). Compared with the low-stable pattern, adjusted HRs were 0.69(0.57–0.83) for the moderate-increasing pattern, 0.66(0.56–0.78) for the moderate-decreasing pattern, 0.43(0.37–0.51) for the high-stable I pattern, and 0.22(0.18–0.28) for the high-stable II pattern, after adjustment for age, sex, education background, drinking status, income level, high-sensitivity C-reactive protein concentrations, estimated glomerular filtration rate, and heart rate.

**Table 2:** Adjusted HRs and 95% CIs for incidence of CVD according to the trajectory of CVHS from 2006 to 2010

	Low-stable	Moderate-increasing	Moderate-decreasing	High-stable I	High-stable II
CVD					
Event/Total	233/2835	233/3492	453/7526	713/17,135	108/6982
Incidence rates*	8.71(7.66–9.90)	7.05(6.20–8.01)	6.33(5.77–6.94)	4.34(4.03–4.67)	1.58(1.31–1.91)
Model 1	Reference	0.71(0.59–0.85)	0.66(0.56–0.77)	0.43(0.37–0.51)	0.22(0.17–0.27)
Model 2	Reference	0.70(0.59–0.84)	0.66(0.56–0.77)	0.44(0.38–0.51)	0.22(0.18–0.28)
Model 3	Reference	0.69(0.57–0.83)	0.66(0.56–0.78)	0.43(0.37–0.51)	0.22(0.18–0.28)
Stroke					
Event/Total	181/2835	183/3492	376/7526	587/17,135	94/6982
Incidence rates*	6.70(5.79–7.75)	5.49(4.75–6.35)	5.22(4.72–5.78)	3.56(3.28–3.86)	1.38(1.12–1.68)
Model 1	Reference	0.72(0.59–0.88)	0.70(0.59–0.84)	0.46(0.39–0.55)	0.24(0.19–0.31)
Model 2	Reference	0.72(0.58–0.88)	0.71(0.59–0.85)	0.47(0.39–0.55)	0.25(0.19–0.32)
Model 3	Reference	0.71(0.58–0.88)	0.71(0.59–0.85)	0.47(0.39–0.56)	0.25(0.19–0.33)
MI					
Event/Total	56/2835	51/3492	84/7526	134/17,135	14/6982
Incidence rates*	2.03(1.56–2.64)	1.51(1.15–1.98)	1.15(0.93–1.42)	0.80(0.68–0.95)	0.20(0.12–0.34)
Model 1	Reference	0.66(0.45–0.96)	0.53(0.37–0.74)	0.36(0.26–0.50)	0.14(0.08–0.25)
Model 2	Reference	0.63(0.43–0.92)	0.53(0.37–0.74)	0.35(0.26–0.49)	0.13(0.07–0.24)
Model 3	Reference	0.58(0.39–0.86)	0.52(0.37–0.73)	0.34(0.24–0.47)	0.13(0.07–0.23)

Abbreviations: CI, confidence interval; CVD, cardiovascular disease; CVHS, cardiovascular health score; HR, hazard ratio

*Cases per 1000 person-years

Model 1 Adjusted for age and sex

Model 2 Included covariates in model 1 and education background (primary, middle or high school, college or above), income level (< 800, or ≥ 800, Chinese yuan/mo)

Model 3 Included covariates in model 2 and high-sensitivity C-reactive protein concentrations, estimated glomerular filtration rate, and heart rate

**Fig. 2:**
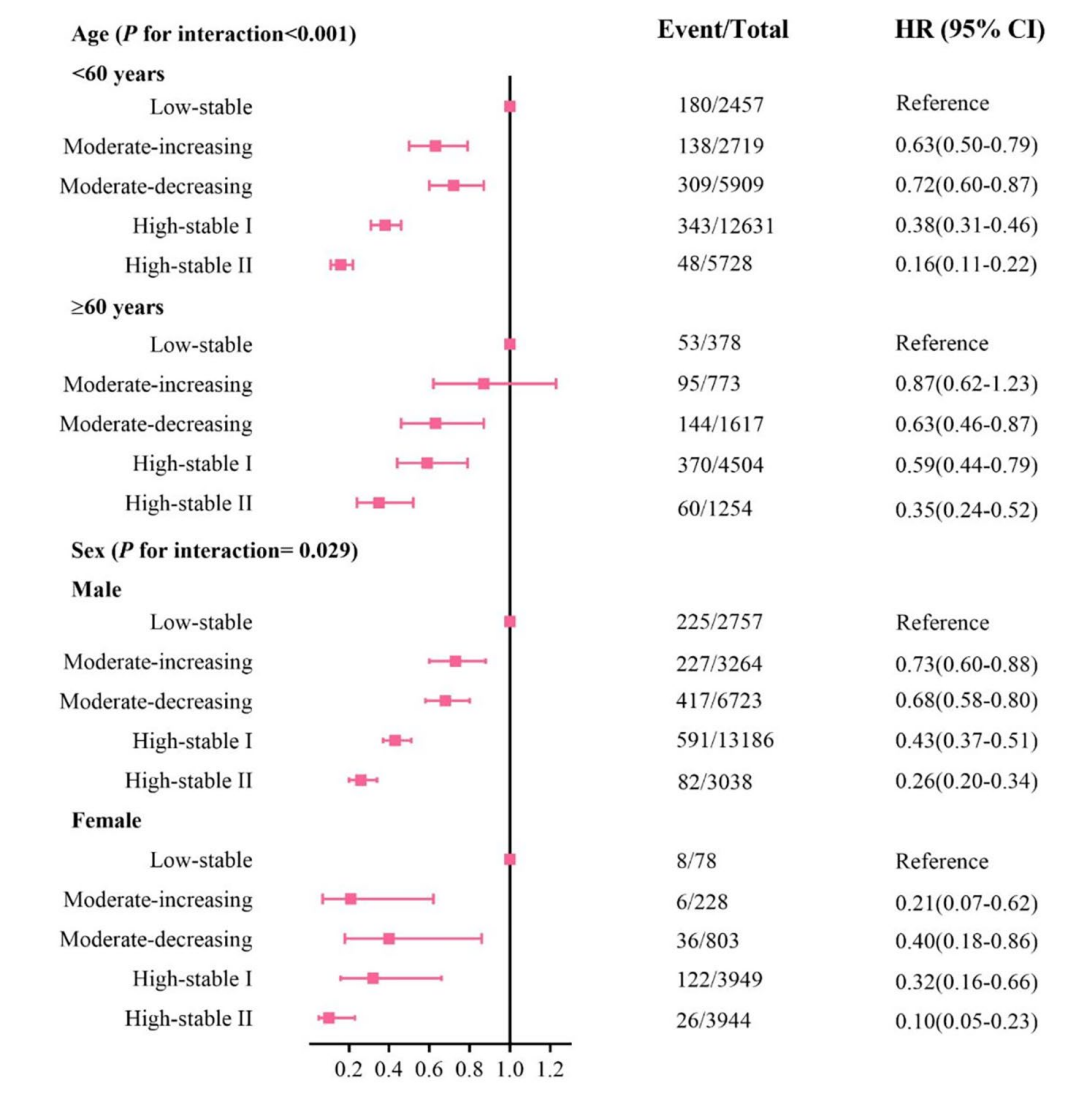
HRs and 95% CIs for stratified analysis of CVD according to the trajectory of cardiovascular health score from 2006 to 2010 Abbreviations: CI, confidence interval; HR, hazard ratio. All models were adjusted for age, sex, education background, drinking status, income level, high-sensitivity C-reactive protein concentrations, estimated glomerular filtration rate, and heart rate

We observed significant interactions between age and sex and CVHS trajectory (*P* for interaction < 0.05). The association was stronger in participants aged < 60 years old than in those aged > 60 years old except for the moderate-decreasing pattern, with adjusted HR of 0.63(0.50–0.79) vs. 0.87(0.62–1.23) for the moderate-increasing pattern, 0.38(0.31–0.46) vs. 0.59(0.44–0.79) for the high-stable I pattern, and 0.16(0.11–0.22) vs. 0.35(0.24–0.52) for the high-stable II pattern. Furthermore, a lower risk for CVD was also observed in females than in those males, with adjusted HR of 0.21(0.07–0.62) vs. 0.73(0.60–0.88) for the moderate-increasing pattern, 0.40(0.18–0.86) vs. 0.68(0.58–0.80) for the moderate-decreasing pattern, 0.32(0.16–0.66) vs. 0.43(0.37–0.51) for the high-stable I pattern, and 0.10(0.05–0.23) vs. 0.26(0.20–0.33) for the high-stable II pattern (Fig. 2). In the sensitivity analysis, after further adjustment for CVHS in the 2006 or 2010 wave, antihypertensive medication, and lipide-lowering medication, the association between CVHS trajectory and CVD risk did not materially change (Supplemental Table S3-S4). Moreover, the results of excluding participants losing to follow-up, and the results of eliminating CVD events in the first two years of follow-up were similar to the primary results (Supplemental Table S5-S6).

## Discussions

In this prospective study of 37,970 participants without diabetes, we identified five distinct CVHS trajectory patterns associated with altered CVD risk over four serial years utilizing trajectory modeling. The primary results showed that participants free of diabetes with the highest CVHS were associated with a 78% lower risk of incident CVD compared with the low-stable CVHS, independent of the baseline CVHS. With the improvement in CVHS over time, the risk of CVD decreased. In addition, stratified by age and sex, a positive association between CVHS trajectory and incident CVD was more pronounced among those aged < 60 years old and females.

The present analyses showed that non-diabetic participants with higher CVHS had a lower risk of CVD than their counterparts with lower CVHS. Previous studies, which were generally based on an assessment of baseline CVHS, found that AHA-defined health metrics were significantly and inversely associated with the risk of CVD events [29, 30]. Moreover, considering the regression dilution, studies based on repeated measures of cardiovascular health metrics could contribute to the true association between CVHS and CVD risk. Wu et al. [11]. Investigated the trajectory of CVHS over four years were associated with altered CVD risk in a large, prospective cohort including 74,701 Chinese adults. Huang et al. identified five distinct CVHS patterns and the highest CVHS had a 76% lower risk of incident MI among hypertensive patients [12]. Likewise, our study also showed the association between 5 distinct CVHS patterns and CVD risk; the highest CVHS was associated with a 78% lower risk of incident CVD. To our knowledge, no prior studies have examined whether CVHS trajectories are related to incident CVD in people without diabetes. However, recent studies suggested that the changes in smoking status [31], fasting blood glucose [19], blood pressure [32], and lipid levels [28] correlated to altered CVD risk, respectively, that smoking cessation, low concentration of fasting blood glucose, enhanced systolic blood pressure reduction, and reduction of TC/HDL-C ratio decreased the risk of CVD. These results from CVHS components indirectly support our findings.

In the age and sex-stratified analysis, the positive associations between CVHS trajectory and incident CVD were observed with statistical significance. Aging is one of the most important risk factors in the development of CVD [32]. Pooled data from 7 cohort studies demonstrated that a higher prevalence of worse CVHS in older age groups across all sex and race groups reinforced the idea. Declining CVHS with age was driven primarily by age-related physiologic risk factors like blood pressure, cholesterol, and glucose, but ideal levels of behavioral factors like physical activity and diet were also less prevalent [33]. It is attractive to consider the possibility of improving overall cardiovascular health status in young and middle-aged adults [11]. Our results also corroborate previous findings that a lower risk for CVD was observed in females in the CVHS trajectory rather than in males of the same category. Epidemiological studies have shown that the prevalence of coronary heart disease is higher in men within each age stratum [34], and the explanation for sex differences may relate to differences in thrombotic and fibrinolytic activity or differences in the clinical presentation of coronary heart disease [35]. The mechanisms underpinning sex differences in CVD risk should also consider differences in risk factor profiles across the life course [33].

Our study added to the database suggesting that maintaining a higher CVHS reduced CVD risk in the non-diabetic population. Diabetes is an important risk factor for CVD, and reducing the incidence of diabetes may reduce CVD’s burden. As the proposal of primordial prevention, it makes sense that identify the risk factors and persons at risk for CVD before the development of overt diabetes [7]. Hu et al. show that just a moderately healthy diet, physical activity, and weight control alone might prevent most new cases of diabetes [36] but only a few people have achieved favorable cardiovascular health metrics [37]. We also observed that people who maintained their high CVHS throughout the study period were at lower CVD risk than those who started with relatively lower CVHS and improved later in life, highlighting the importance of having a favorable CVHS as early as possible.

One of the study’s strengths is the association between the trajectory of CVHS and CVD risk that we found in the non-diabetic population through a 4-year assessment and 10-year follow-up. The prospective design and high follow-up rates of the Kailuan study minimize the potential recall bias and loss of follow-up, making it possible to capture a large number of CVD incidence events. However, this study also has several limitations. Firstly, this is not a national representative sample. The cohort consisted of Chinese adults from the Kailuan community in north China with a relatively small female population (23.2%), limiting the generalization of the results to generalizability. Secondly, despite the large sample size, the CVHS trajectory was based on 3 measurements over 4 years. Defining trajectory over time in such a short period is not optimal. Thirdly, we used salt intake as a proxy for the dietary component of the CVHS during our analysis, and the other information related to diet was not available until 2014. In subsequent dietary studies, we found a strong association between higher salt intake and lower healthy diet scores [11, 38]. Although, excessive salt intake was strongly associated with the development of CVD [39], the results still need to be interpreted with caution.

## Conclusions

Maintaining a prolonged high CVHS presented by the overall cardiovascular health status would lower the CVD risk among non-diabetic participants. When stratified by age and sex, the association was higher in younger adults and females. Therefore, implementing proactive primary prevention strategies to optimize cardiovascular health behaviors and risk factors is of great importance among non-diabetic participants.

## Electronic supplementary material

Below is the link to the electronic supplementary material.

Supplementary Material 1

## Data Availability

The datasets used and/or analyzed during the current study are not publicly available but are available from the corresponding author at reasonable request.

## Abbreviations

CVD: Cardiovascular disease
CVHS: Cardiovascular health score
HR: Hazard ratio
CI: Confidence interval
