# Supplementary material for: Association of cardiovascular health score trajectory and risk of subsequent cardiovascular disease in non-diabetic population: a cohort study
Source: BMC Public Health. 2023 Jun 1;23:1043. doi: 10.1186/s12889-023-15569-z (PMC10233877; doi:10.1186/s12889-023-15569-z)
Supplement: Supplementary file 1 — Supplementary Material 1 [file 12889_2023_15569_MOESM1_ESM.docx]

**Supplementary materials**

**Supplemental Figure S1.** Flow Chart of Subject Selection Process

**Supplemental Table S1.** Characteristics of participants included and excluded.

**Supplemental Table S2.** Characteristics of participants losing to follow-up.

**Supplemental Table S3.** HRs and 95% CIs for incidence of CVD according to the trajectory of CVHS after further adjustment.

**Supplemental Table S4.** HRs and 95% CIs for incidence of CVD according to the trajectory of CVHS with adjustment for antihypertensive medication and lipide-lowering medication.

**Supplemental Table S5.** HRs and 95% CIs for incidence of CVD according to the trajectory of CVHS excluding participants losing to follow-up.

**Supplemental Table S6.** HRs and 95% CIs for incidence of CVD according to the trajectory of CVHS from 2006 to 2010: excluding outcomes occurring during the first 2 years of follow-up.

49013 participants without diabetes between 2006 and 2010 wave were enrolled

1976 participants who developed cardiovascular disease, or death in or before 2010 were excluded

5350 participants who missed data on cardiovascular heath metric in 2008 or 2010 were excluded

37970 participants included for analysis

1148 participants with cardiovascular diseases in 2006 were excluded

2569 participants with missing data on cardiovascular heath metrics in 2006 were excluded

**Supplemental Figure S1.** Flow Chart of Subject Selection Process.

**Supplemental Table S1**. Characteristics of participants included and excluded.

|  | Total | Excluded | Included |
| --- | --- | --- | --- |
| No. of participants | 49013 | 11043 | 37970 |
| Age |  |  |  |
| Mean (SD), y | 52.7±12.1 | 56.6±12.7 | 51.6±11.8 |
| Missing (%) | 150(0.3) | 150(1.4) | - |
| Sex |  |  |  |
| Female | 11901(24.3) | 2899(26.2) | 9002(23.7) |
| Men | 37112(75.7) | 8144(73.8) | 28968(76.3) |
| Missing (%) | - | - | - |
| Heart rate |  |  |  |
| Mean (SD), beats/min | 72.7±10.1 | 72.0±10.4 | 73.0±10.0 |
| Missing (%) | 54.0(0.1) | 54.0(0.5) | - |
| Hs-CRP |  |  |  |
| Median (IQR), mg/L | 1.0(0.5-2.3) | 1.1(0.6-2.7) | 1.0(0.5-2.3) |
| Missing (%) | 1291(2.6) | 1291(11.7) | - |
| eGFR |  |  |  |
| Median (IQR), mL/min/1.73 m^2^ | 87.3(71.9-99.7) | 93.5(78.2-104.0) | 92.7(76.3-104.9) |
| Missing (%) | 482(1.0) | 482(4.4) | - |
| Education background |  |  |  |
| Primary | 3374(6.9) | 1020(9.2) | 2354(6.2) |
| Middle/high school | 39208(80.0) | 8125(73.6) | 31083(81.9) |
| College or above | 6425(13.1) | 1892(17.2) | 4533(11.9) |
| Missing (%) | 6(0.1) | 6(0.1) | - |
| Drinking status |  |  |  |
| No | 31775(65.1) | 7415(68.3) | 24360(64.2) |
| Yes | 17050(34.9) | 3440(931.7) | 13610(35.8) |
| Missing (%) | 188(0.4) | 188(1.7) | - |
| Income level |  |  |  |
| < 800, Chinese yuan/mo | 18615(49.0) | - | 18615(49.0) |
| ≥ 800, Chinese yuan/mo | 19355(51.0) | - | 19355(51.0) |
| Missing (%) | 11043(22.5) | 11043(100.0) | - |
| Antihypertensive medications |  |  |  |
| No | 38965(79.5) | 7739(70.1) | 31226(82.2) |
| Yes | 10048(20.5) | 3304(29.9) | 6744(17.8) |
| Missing (%) | - | - | - |
| Lipid-lowering medications |  |  |  |
| No | 48253(98.5) | 10742(97.3) | 37511(98.8) |
| Yes | 760(1.6) | 301(2.7) | 459(1.2) |
| Missing (%) | - | - | - |

Abbreviations: CVHS, cardiovascular health score; eGFR, estimated glomerular filtration rate; Hs-CRP, hypersensitive c-reactive protein.

**Supplemental Table S2**. Characteristics of participants losing to follow-up.

|  | Total | Followed up | Losing to follow-up |
| --- | --- | --- | --- |
| No. of participants | 37970 | 36741 | 1229 |
| Age, mean (SD), y | 51.6±11.8 | 51.5±11.8 | 54.7±13.0 |
| Men | 28968(76.3) | 27949(76.1) | 1019(82.9) |
| Heart rate, mean (SD), beats/min | 73.0±10.0 | 73.0±10.0 | 73.2±10.5 |
| Hs-CRP, median (IQR), mg/L | 1.0(0.5-2.3) | 1.0(0.5-2.3) | 1.0(0.4-2.3) |
| eGFR, median (IQR), mL/min/1.73 m^2^ | 92.3(75.3-103.9) | 92.7(76.3-104.9) | 92.1(74.7-103.0) |
| CVHS in 2006, mean (SD), points | 8.8±1.9 | 8.8±1.9 | 8.7±2.0 |
| CVHS in 2010, mean (SD), points | 8.4±2.0 | 8.4±2.0 | 8.3±2.1 |
| Education background |  |  |  |
| Primary | 2354(6.2) | 2253(6.1) | 101(8.2) |
| Middle/high school | 31083(81.9) | 30172(82.1) | 911(74.1) |
| College or above | 4533(11.9) | 4316(11.8) | 217(17.7) |
| Drinking status |  |  |  |
| No | 24360(64.2) | 23568(64.2) | 792(64.4) |
| Yes | 13610(35.8) | 13173(35.9) | 437(35.6) |
| Income level |  |  |  |
| < 800, Chinese yuan/mo | 18615(49.0) | 18051(49.1) | 564(45.9) |
| ≥ 800, Chinese yuan/mo | 19355(51.0) | 18690(50.9) | 665(54.1) |
| Antihypertensive medications |  |  |  |
| No | 31226(82.2) | 30253(82.3) | 973(79.2) |
| Yes | 6744(17.8) | 6488(17.7) | 256(20.8) |
| Lipid-lowering medications |  |  |  |
| No | 37511(98.8) | 36295(98.8) | 1216(98.9) |
| Yes | 459(1.2) | 446(1.2) | 13(1.1) |

Abbreviations: CVHS, cardiovascular health score; eGFR, estimated glomerular filtration rate; Hs-CRP, hypersensitive c-reactive protein.

**Supplemental Table S3.** HRs and 95% CIs for incidence of CVD according to trajectories of CVHS after further adjustment

|  | Low-stable | Moderate-increasing | Moderate-decreasing | High-stable I | High-stable II |
| --- | --- | --- | --- | --- | --- |
| CVD |  |  |  |  |  |
| Event/Total | 233/2835 | 233/3492 | 453/7526 | 713/17135 | 108/6982 |
| Incidence rates* | 8.71(7.66-9.90) | 7.05(6.20-8.01) | 6.33(5.77-6.94) | 4.34(4.03-4.67) | 1.58(1.31-1.91) |
| Further adjustment for CVHS in 2006 | Reference | 0.72(0.59-0.87) | 0.73(0.60-0.88) | 0.50(0.40-0.62) | 0.28(0.20-0.38) |
| Further adjustment for CVHS in 2010 | Reference | 0.83(0.66-1.04) | 0.71(0.60-0.84) | 0.53(0.43-0.66) | 0.31(0.22-0.43) |
| Stroke |  |  |  |  |  |
| Event/Total | 181/2835 | 183/3492 | 376/7526 | 587/17135 | 94/6982 |
| Incidence rates* | 6.70(5.79-7.75) | 5.49(4.75-6.35) | 5.22(4.72-5.78) | 3.56(3.28-3.86) | 1.38(1.12-1.68) |
| Further adjustment for CVHS in 2006 | Reference | 0.75(0.61-0.92) | 0.79(0.64-0.98) | 0.54(0.43-0.69) | 0.32(0.23-0.45) |
| Further adjustment for CVHS in 2010 | Reference | 0.80(0.62-1.03) | 0.74(0.61-0.89) | 0.53(0.42-0.68) | 0.31(0.22-0.44) |
| Myocardial infarction |  |  |  |  |  |
| Event/Total | 56/2835 | 51/3492 | 84/7526 | 134/17135 | 14/6982 |
| Incidence rates* | 2.03(1.56-2.64) | 1.51(1.15-1.98) | 1.15(0.93-1.42) | 0.80(0.68-0.95) | 0.20(0.12-0.34) |
| Further adjustment for CVHS in 2006 | Reference | 0.60(0.40-0.90) | 0.57(0.38-0.86) | 0.38(0.24-0.61) | 0.15(0.07-0.33) |
| Further adjustment for CVHS in 2010 | Reference | 0.91(0.56-1.49) | 0.62(0.43-0.89) | 0.57(0.35-0.90) | 0.28(0.13-0.62) |

Abbreviations: CVD, cardiovascular disease; CVHS, cardiovascular health score; HR, hazard ratio; CI, confidence interval.

*Cases per 1000 person-years.

Further adjustment was based on the adjustment for age, sex, education background, drinking status, income level, high-sensitivity C-reactive protein concentrations, estimated glomerular filtration rate, and heart rate.

**Supplemental Table S4.** HRs and 95% CIs for incidence of CVD according to the trajectory of CVHS with adjustment for antihypertensive medication and lipide-lowering medication.

|  | Low-stable | Moderate-increasing | Moderate-decreasing | High-stable I | High-stable II |
| --- | --- | --- | --- | --- | --- |
| CVD |  |  |  |  |  |
| Event/Total | 233/2835 | 233/3492 | 453/7526 | 713/17135 | 108/6982 |
| Incidence rates* | 8.71(7.66-9.90) | 7.05(6.20-8.01) | 6.33(5.77-6.94) | 4.34(4.03-4.67) | 1.58(1.31-1.91) |
| Model | Reference | 0.74(0.62-0.89) | 0.72(0.61-0.84) | 0.50(0.43-0.59) | 0.28(0.22-0.35) |
| Stroke |  |  |  |  |  |
| Event/Total | 181/2835 | 183/3492 | 376/7526 | 587/17135 | 94/6982 |
| Incidence rates* | 6.70(5.79-7.75) | 5.49(4.75-6.35) | 5.22(4.72-5.78) | 3.56(3.28-3.86) | 1.38(1.12-1.68) |
| Model | Reference | 0.78(0.63-0.96) | 0.78(0.65-0.93) | 0.56(0.47-0.67) | 0.33(0.25-0.43) |
| Myocardial infarction |  |  |  |  |  |
| Event/Total | 56/2835 | 51/3492 | 84/7526 | 134/17135 | 14/6982 |
| Incidence rates* | 2.03(1.56-2.64) | 1.51(1.15-1.98) | 1.15(0.93-1.42) | 0.80(0.68-0.95) | 0.20(0.12-0.34) |
| Model | Reference | 0.59(0.40-0.87) | 0.53(0.37-0.74) | 0.35(0.25-0.48) | 0.13(0.07-0.24) |

Abbreviations: CVD, cardiovascular disease; CVHS, cardiovascular health score; HR, hazard ratio; CI, confidence interval.

*Cases per 1000 person-years.

The model was adjusted for age, sex, education background, drinking status, income level, high-sensitivity C-reactive protein concentrations, estimated glomerular filtration rate, heart rate, antihypertensive medications, and lipide-lowering medications.

**Supplemental Table S5**. HRs and 95% CIs for incidence of CVD according to the trajectory of CVHS excluding participants losing to follow-up (36741).

|  | Low-stable | Moderate-increasing | Moderate-decreasing | High-stable I | High-stable II |
| --- | --- | --- | --- | --- | --- |
| CVD |  |  |  |  |  |
| Event/Total | 233/2751 | 233/3367 | 453/7267 | 713/16563 | 108/6793 |
| Incidence rates* | 8.98(7.90-10.22) | 7.32(6.44-8.33) | 6.56(5.98-7.19) | 4.49(4.18-4.84) | 1.63(1.35-1.96) |
| Model | Reference | 0.70(0.58-0.84) | 0.66(0.56-0.78) | 0.43(0.37-0.51) | 0.22(0.17-0.28) |
| Stroke |  |  |  |  |  |
| Event/Total | 181/2751 | 183/3367 | 376/7267 | 587/16563 | 94/6793 |
| Incidence rates* | 6.91(5.97-7.99) | 5.70(4.94-6.59) | 5.41(4.89-5.99) | 3.69(3.40-4.00) | 1.41(1.15-1.73) |
| Model | Reference | 0.72(0.58-0.89) | 0.71(0.59-0.85) | 0.47(0.39-0.56) | 0.25(0.19-0.33) |
| Myocardial infarction |  |  |  |  |  |
| Event/Total | 56/2751 | 51/3367 | 84/7267 | 134/16563 | 14/6793 |
| Incidence rates* | 2.10(1.61-2.72) | 1.57(1.19-2.06) | 1.19(0.96-1.47) | 0.83(0.70-0.99) | 0.21(0.12-0.35) |
| Model | Reference | 0.59(0.40-0.86) | 0.52(0.37-0.73) | 0.34(0.24-0.47) | 0.12(0.07-0.23) |

Abbreviations: CVD, cardiovascular disease; CVHS, cardiovascular health score; HR, hazard ratio; CI, confidence interval.

*Cases per 1000 person-years.

The model was adjusted for age, sex, education background, drinking status, income level, high-sensitivity C-reactive protein concentrations, estimated glomerular filtration rate, and heart rate.

**Supplemental Table S6.** HRs and 95% CIs for incidence of CVD according to trajectories of CVHS from 2006 to 2010: excluding outcomes occurring during first 2 years of follow-up (40519).

|  | Low-stable | Moderate-increasing | Moderate-decreasing | High-stable I | High-stable II |
| --- | --- | --- | --- | --- | --- |
| CVD |  |  |  |  |  |
| Event/Total | 180/2782 | 180/3439 | 350/7423 | 572/16993 | 87/6961 |
| Incidence rates* | 6.75(5.84-7.82) | 5.46(4.72-6.32) | 4.90(4.42-5.45) | 3.49(3.21-3.79) | 1.28(1.03-1.57) |
| Model | Reference | 0.69(0.56-0.86) | 0.66(0.55-0.79) | 0.45(0.38-0.54) | 0.24(0.18-0.31) |

Abbreviations: CVD, cardiovascular disease; CVHS, cardiovascular health score; HR, hazard ratio; CI, confidence interval.

*Cases per 1000 person-years.

The model was adjusted for age, sex, education background, drinking status, income level, high-sensitivity C-reactive protein concentrations, estimated glomerular filtration rate, and heart rate.
